# Functional annotation of a divergent genome using sequence and structure-based similarity

**DOI:** 10.1186/s12864-023-09924-y

**Published:** 2024-01-02

**Authors:** Dennis Svedberg, Rahel R. Winiger, Alexandra Berg, Himanshu Sharma, Christian Tellgren-Roth, Bettina A. Debrunner-Vossbrinck, Charles R. Vossbrinck, Jonas Barandun

**Affiliations:** 1 Department of Molecular Biology, The Laboratory for Molecular Infection Medicine Sweden (MIMS), Science for Life Laboratory, Umeå Centre for Microbial Research (UCMR), Umeå University, Umeå, 90187 Sweden; 2 Department of Medical Biochemistry and Biophysics, Umeå University, Umeå, 90736 Sweden; 3 Science for Life Laboratory, Department of Immunology, Genetics and Pathology, Uppsala University, Uppsala, Sweden; 4 Department of Math/Science, Gateway Community College, 20 Church Street, New Haven, CT 06510 USA; 5 Department of Environmental Science, Connecticut Agricultural Experiment Station, New Haven, CT 06504 USA

**Keywords:** Functional annotation, Genome, Microsporidia, Polar tube proteins, Ricin B lectins, Structural similarity, *Vairimorpha necatrix*

## Abstract

**Background:**

Microsporidia are a large taxon of intracellular pathogens characterized by extraordinarily streamlined genomes with unusually high sequence divergence and many species-specific adaptations. These unique factors pose challenges for traditional genome annotation methods based on sequence similarity. As a result, many of the microsporidian genomes sequenced to date contain numerous genes of unknown function. Recent innovations in rapid and accurate structure prediction and comparison, together with the growing amount of data in structural databases, provide new opportunities to assist in the functional annotation of newly sequenced genomes.

**Results:**

In this study, we established a workflow that combines sequence and structure-based functional gene annotation approaches employing a ChimeraX plugin named ANNOTEX (Annotation Extension for ChimeraX), allowing for visual inspection and manual curation. We employed this workflow on a high-quality telomere-to-telomere sequenced tetraploid genome of *Vairimorpha necatrix.* First, the 3080 predicted protein-coding DNA sequences, of which 89% were confirmed with RNA sequencing data, were used as input. Next, ColabFold was used to create protein structure predictions, followed by a Foldseek search for structural matching to the PDB and AlphaFold databases. The subsequent manual curation, using sequence and structure-based hits, increased the accuracy and quality of the functional genome annotation compared to results using only traditional annotation tools. Our workflow resulted in a comprehensive description of the *V. necatrix* genome, along with a structural summary of the most prevalent protein groups, such as the ricin B lectin family. In addition, and to test our tool, we identified the functions of several previously uncharacterized *Encephalitozoon cuniculi* genes.

**Conclusion:**

We provide a new functional annotation tool for divergent organisms and employ it on a newly sequenced, high-quality microsporidian genome to shed light on this uncharacterized intracellular pathogen of Lepidoptera. The addition of a structure-based annotation approach can serve as a valuable template for studying other microsporidian or similarly divergent species.

**Supplementary Information:**

The online version contains supplementary material available at 10.1186/s12864-023-09924-y.

## Background

Traditional functional gene annotation relies on sequence similarity between the studied species and previously characterized genes from other model organisms [1–5]. However, sequence similarity can be lost over large evolutionary distance [6, 7] and, thus, can be very low among highly divergent species [8–10]. Microsporidia are highly divergent, fungal-like parasites with streamlined and rapidly evolving genomes [9, 11–13]. As obligate intracellular pathogens, they have been found to infect hosts from almost all animal taxa, including humans [14, 15]. In addition to their medical relevance [16], microsporidia infections of our two most important domesticated insects, silkworms (infected by *Nosema bombycis*) and honeybees (infected by *Vairimorpha ceranae* and *Vairimorpha apis*) cause significant economic losses. Loss of pollination by honeybees and other pollinator species due to microsporidial infections pose a potential threat to global food supplies [17–19]. Thus, further analyses of Vairimorphan genomic repertoire and virulence mechanisms are needed to combat microsporidiosis and save important pollinators. Microsporidia develop inside a host cell and are spread to other hosts through an external spore stage. The obligate intracellular nature of microsporidia and the adaptation to this lifestyle has led to the loss of many proteins or sometimes whole biosynthetic pathways [11, 20–22]. In addition, microsporidia have shortened not only many of their genes [9] but have also reduced intergenic regions. *Encephalitozoon cuniculi*, for example, which is commonly infecting rodents and one of the most studied microsporidian species [23], reduced the intergenic regions to an average of 107 bp, leaving them with unusually compacted genomes [8, 9, 24, 25]. To date, the most extreme case of eukaryotic genome miniaturization is found in the human parasite *Encephalitozoon intestinalis* at 2.3 Mb with only 1934 densely packed genes [5]. Despite the reductive evolution, microsporidia have evolved species-specific properties, including a unique and highly specialized polar tube (PT) [26] for transferring the sporoplasm of the microsporidian to the host cell. The interaction with the host cell is, amongst others, established through the binding of polar tube protein 4 (PTP4) [27], spore wall proteins (SWPs) [28, 29], and ricin B lectins (RBLs) [30–32]. RBL proteins are a group of carbohydrate-binding proteins that were reported to have expanded in the microsporidian order of Nosematida and are important for host-cell invasion and thus pathogenicity [30–32]. Consistent with the high sequence diversity of microsporidian genomes, RBL protein sequences were reported to have very low sequence similarity [31, 32]. Taken together, the distinctive development of microsporidia, which involves genome reduction, species-specific specialization, and accelerated evolutionary rate, has resulted in significant sequence divergence [33].

This divergence observed in microsporidia poses several challenges for traditional sequence-based annotation methods: First, early branching in the fungal kingdom creates a great evolutionary distance to fungal model organisms resulting in diminished sequence similarity [34]. Second, the accelerated genome evolution, employing gene deletions, mutations, and shortenings as well as enrichments through gene duplications and horizontal gene transfer (from host organisms and bacteria) [11, 35, 36], shaped a highly divergent clade, not only compared to distantly related organisms but also within the clade itself. Lastly, by optimizing the requirements to infect and thrive in their host [22, 37], microsporidia have evolved their own specific set of core genes, which may not exist in other well-studied fungal organisms, such as *Saccharomyces cerevisiae*. In addition, low sequence similarity for universally conserved genes often makes it difficult to find and confirm their homologs in microsporidia.

Unlike primary sequences, protein structures remain more conserved over time [38, 39] which is essential to retain their functions [40]. Proteins with similar functions generally maintain a structural similarity [38, 41]. The gold standard for functional protein annotation is experimental characterization, including molecular, biochemical, and biophysical analyses. However, the experimental characterization of microsporidian proteins is often not achievable as both culturing and genetic manipulation of microsporidia are challenging. Furthermore, the divergent nature of microsporidian genes, AT-rich genomes, a large fraction of exported disulfide-containing proteins, and codon bias, make it difficult to use typical model organisms such as *Escherichia coli* or *S. cerevisiae* for protein production. Therefore, experimentally verified functional protein annotations lag far behind the amount of sequencing data [42]. However, recent advances in protein structure prediction provide an improved basis for structure-based functional annotations [43–45]. Local, optimized software versions, such as ColabFold [46], facilitate creating proteome-wide structure predictions, and Foldseek [47], a fast structural aligner, can now be used to search through databases consisting of millions of structures within seconds.

In this study, we sequenced genomic DNA (gDNA) and total RNA from germinated *Vairimorpha necatrix* (*V. necatrix*) spores, revealing a tetraploid genome with 12 complete chromosomes and 2971 genes. The Benchmarking Universal Single-Copy Orthologs (BUSCO) analysis [48] showed a high completeness score of > 95%. We combined structural and sequence-based similarity to functionally annotate protein-encoding genes of *V. necatrix*. For this, we developed the ANNOTEX plugin for ChimeraX, a next-generation molecular visualization program for the interactive visualization and analysis of molecular structures and related data (https://www.cgl.ucsf.edu/chimerax/). ANNOTEX allows to visually inspect every structural annotation match and curate the best hits. Using this approach, we enhanced the prediction of gene function by 10.36% compared to when only relying on sequence-based similarity. Further, we found additional, previously unidentified members of the expanded RBL family and show that PTP4, PTP5 and PTP6 are members of the RBL family.

## Results & discussion

### The genome architecture of ***V. necatrix***

**Fig. 1:**
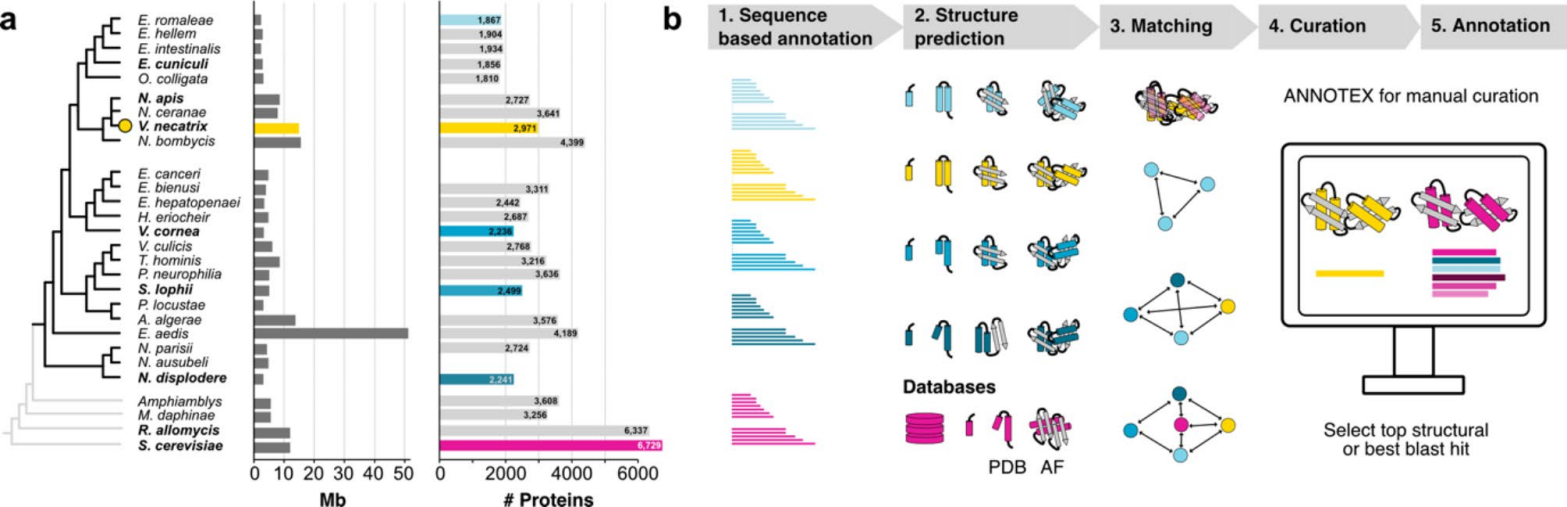
Functional annotation of *V. necatrix* genes using structure-prediction and sequence-based comparative analyses. **a**) A phylogenetic tree based on [25] with 24 microsporidian species, and 3 outgroup species plus *S. cerevisiae* (grey branches). The bar graphs show the respective genome sizes and the number of proteins used (colored) and folded for our structural comparison. **b**) Schematic pipeline of our structural similarity approach, from protein structure prediction with ColabFold (v1.5.2) to structural matching using Foldseek (v5-53465f0), followed by a manual curation step with ANNOTEX that includes a comparison of sequence and structure-based hits to achieve a high-quality functional annotation

We propagated *V. necatrix* in the corn earworm *Helicoverpa zea*, followed by the isolation of highly pure mature spores, which were used for gDNA extraction. The gDNA was sent to the National Genomics Infrastructure Uppsala Genome Center for PacBio *de novo* sequencing and assembly (Table 1). The assembly with a standard diploid assembler resulted in two haplotypes with two sets of chromosomes each. This tetraploid nature of *V. necatrix* spores is expected as the organism is diplokaryotic [49, 50]. This contrasts with many other microsporidian species, which have been reported to be strictly monokaryotic throughout their life cycle (such as *E. cuniculi*). However, a recent study [51] confirmed the identification of tetraploid species within the Nosematida clade, to which *V. necatrix* belongs. Each pseudo-haplotype (called 1–4) consists of 12 chromosomes. The pentanucleotide repeat TAACC and its reverse complement were manually identified as signatures of telomeric repeats. The analysis with telomeric-identifier found telomeric repeats on 42 of the 48 contig ends, confirming two complete telomere-to-telomere haplotypes (Supplementary Fig. 1). The assembled pseudo-haplotypes are 15.3, 15.1, 14.8, and 14.7 Mb in size, resulting in a total assembly of 59.9 Mb. The variation in the pseudo-haplotypes’ length is only marginally influenced, for example, by the missing telomeres of single chromosomes (max. 20 kb per missing telomere). Overall, the four pseudo-haplotypes share a sequence identity of 96% as assessed by dnadiff [52]. The genome has an overall GC content of 28.3%, and repeated regions make up roughly 50%. To date, assembled microsporidian genomes range from 2.3 Mb (*E. intestinalis*) [5] to 51.3 Mb (*Edhazardia aedis*) [53], placing *V. necatrix* with an average pseudo-haplotype size of 14.97 Mb among the medium-sized microsporidian genomes (Fig. 1a). We predicted 3080 genes (of which 109 were later annotated as additional transposable elements) using BRAKER [54], resulting in a coding density of 20.8%. This coding density is on the lower end among microsporidia but is typical for species with a medium-sized to large genome [55–57]. In comparison, the *E. cuniculi* genome (only 2.9 Mb) has a coding density of 84%. This genome compaction is a result of gene shortening, overlapping genes, and a shortening of intergenic regions [8, 58]. In the *V. necatrix* genome, however, we only identified three overlapping coding sequences. With a mean length of 3606 bp, the intergenic regions are not as significantly shortened as those of other microsporidians like *E. cuniculi* (107 bp) and *E. intestinalis* (115 bp) [5].

To evaluate the quality and completeness of the *V. necatrix* genome, we used BUSCO [48] with 600 predefined microsporidian-specific genes. The presence or absence of highly conserved genes serves as an indirect measure of the completeness of an assembly [48]. The four pseudo-haplotypes have BUSCO completeness scores from 95.9 to 96.5% (11 missing and 10 fragmented genes) suggesting a complete genome and accurate gene prediction (Table 1). We used RNA sequencing data to further validate the gene predictions. For this, RNA was extracted from *V. necatrix* sporoplasms, immediately after its release through the PT in a process called germination, and sent for sequencing. The obtained RNA reads were subsequently aligned to the predicted genes using STAR [59]. The genes with aligned reads were classified as confirmed (88.7%, or 2732 of 2971 genes and 109 TEs), and those with no aligned reads might either be miss-annotated or not expressed during this early measured time point.

**Table 1:** General features of the *V. necatrix* genome

Haploid genome size (Mb)	15.3 (H1), 15.1 (H2), 14.8 (H3), and 14.7 Mb (H4), Average 14.97
Ploidy	Tetraploid
Repeat content (%)	50.0
GC content (%GC)	28.3
Protein-coding genes	2971
RNA-seq confirmed	88.7%, or 2732/3080 genes
Transposable elements	109
Gene density (genes/kb)	0.20
Mean coding length (bp)	1081
Mean intergenic distance (bp)	3606
Number of overlapping genes	3
rDNA genes (16-23 S/5S)	18 (hap1, 3) − 20 (hap2, 4) / 13
# of genes with signal peptide	372 (128 also have a TM)
# of genes with transmembrane domains	382 (128 also have a SP)
BUSCO scores of haplotypes	95.9–96.5%

### ANNOTEX for functional genome annotation

Due to microsporidia’s divergent nature and the resulting low sequence similarity to proteins in model organisms, many genes’ functions could not be inferred. Similarly, an initial functional annotation of the *V. necatrix* genome with eggNOG [60] and based on sequence similarity, resulted in 65% hypothetical genes. Previous analyses have shown that structure is often more conserved than sequence, and homologs adopt similar folds despite a very low sequence similarity [38]. For divergent organisms such as microsporidia, structural predictions based on multiple sequence alignments may be less accurate due to the potential for reduced sequence similarity and homology. However, for predicted folds with high confidence, a structure-based approach can effectively identify potential function. Therefore, we conducted a comprehensive structure-based, comparative examination to complement the functional annotation of the *V. necatrix* genome.

First, we used ColabFold to predict protein structures for all identified genes in the *V. necatrix* genome and for full proteomes from representative members of the major microsporidian clades (Fig. 1a). The predicted structures were then matched to the AlphaFold Database (AFDB) and Protein Data Bank (PDB), using Foldseek in a one-to-all structure-based search (Fig. 1b). This allowed us to obtain structural similarity scores and top-ranking protein matches. While a structure-based approach can provide complementary functional information on many of the divergent microsporidian proteins, it relies on the quality of the structure prediction and the presence of well-folded domains. Disordered and very small proteins generate only a few structural matches, while ubiquitous domain folds, like short helices, structurally match with many different types of proteins that might be functionally unrelated. Therefore, we concluded that combining a sequence and a structure-based approach, focusing on high-confidence structure predictions (Additional File 1), and including a manual curation step for each protein, is best for the functional gene annotation of a divergent organism. To achieve this, we developed the ChimeraX annotator plugin ANNOTEX that visually combines the results from structural matches (Foldseek top matches from AFDB, PDB, and in this study folded microsporidian proteomes) with eggNOG [60] annotations and the top blast hits (Diamond [61]), while allowing for manual curation (Supplementary Fig. 2, Supplementary Fig. 3). We also displayed transmembrane domain (TMD) and signal-peptide (SP) prediction results in ANNOTEX. We manually curated each protein from *V. necatrix* and updated or complemented the functional annotation. In addition, we used two previous structural studies [62, 63], to annotate the ribosomal and proteasomal genes. The high-quality annotation of these proteins can help to improve and correct the functional annotation of other microsporidian organisms. Further, to show that our functional gene annotations are based on high-confidence structure predictions, we summarized the pLDDT scores of protein structures for hypothetical, uncharacterized and annotated genes (Supplementary Fig. 4b). Most annotated genes have a pLDDT score of more than 70, suggesting good to high quality (Supplementary Fig. 4b, **green violin plot**). The small number of genes with pLDDT scores around 60 were carefully inspected prior to annotating a function and complemented with high quality sequence-based hits.

Shortly after finishing our annotation efforts, the automated annotation tool ProtNLM replaced eggNOG as the standard method for gene function prediction. Hence, we compared our annotation results to those of ProtNLM (Benchmarking of our approach). This allowed us to obtain an additional 229 annotations from ProtNLM for gene functions that our tool suggested to be uncharacterized or hypothetical.

The complete, manually curated annotations, including the pLDDT average scores of all structural predictions to provide a quality measure and Interproscan (5.65-97.0-64-bit) results, can be found in (Additional file 1). Our annotation database, including all predicted structures, are available as Supplementary Data File deposited to Zenodo (10.5281/zenodo.7974739) and ANNOTEX is available on https://github.com/Barandun-Lab/ANNOTEX.

### The annotated genome of ***V. necatrix***

**Fig. 2:**
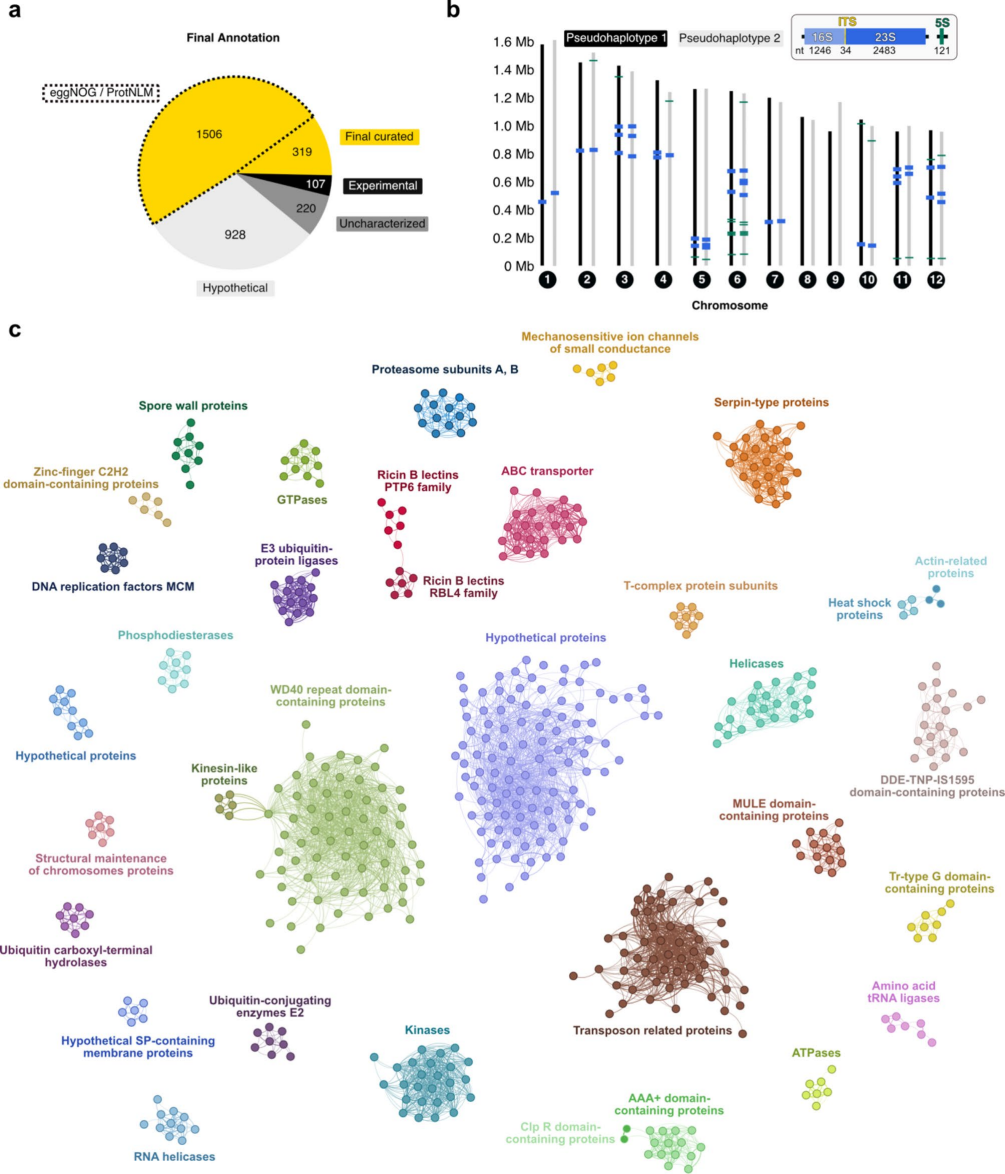
The annotated genome of *V. necatrix* (**a**) Pie chart summarizing the functional annotation output using a combination of sequence and structure-based hits and experimental data. Compared to ProtNLM or eggNOG (yellow, marked by black dashed lines), our complementary approach improved the genome annotation by an additional 319 final curated gene functions, here shown in yellow. Further, 107 experimentally solved protein structures (black) from PDB are listed as structural matches. 220 genes that have homologs in other microsporidia, but are of unknown function, are presented in dark grey. Light grey represents 928 hypothetical *V. necatrix* genes that have no matches to the known genes of other microsporidia. (**b**) Approximate localization of the rDNA genes 16 S/23S (blue) and 5 S (green) on the 12 chromosomes of the two predominant pseud-haplotypes 1 (black) and 2 (grey). The insert depicts one rDNA in shades of blue (light blue for the 16 S, dark blue for the 23 S) and one 5 S gene in green. The internal transcribed spacer (ITS) is shown in yellow. (**c**) Structure-based network of highly abundant protein-fold families encoded by our *V. necatrix* genome. AlphaFold-predicted protein models were analyzed for structural relatedness in a Foldseek all-against-all search. The structural similarity is represented by the TM score which is used as a measure for the protein network graph generated in Gephi (v0.9.2). Each node represents a protein colored according to its fold family. Proteins with inverted surrounding and filling color compared to the main cluster have an additional common domain besides the one unifying the main cluster i.e., Clp R domain-containing proteins and actin(-like) proteins. Connecting lines indicate structural relation of proteins and thicker lines indicate greater structural similarity. PTP6, polar tube protein 6; RBL, ricin B lectin; MCM, minichromosome maintenance; Serpin-type protein, serine-protease inhibitor type protein; MULE domain, Mutator-like elements domain; Tr-type G domain, translation-type guanosine-binding domain; SP, signal peptide; Clp R domain, caseinolytic protease repeat domain; AAA+, ATPases associated with diverse cellular activities

We employed ANNOTEX on all 3080 predicted proteins (number includes some TE missed by RepeatModeler) that were obtained from genes of the *de novo* assembled *V. necatrix* genome. We functionally annotated 1932 proteins in total using combined information from sequence, predicted structural, and available experimental data (Fig. 2a, **Final curated**). Compared to eggNOG and ProtNLM, we were able to annotate an additional 319 genes in the *V. necatrix* genome, excluding the information from experimentally verified proteins. Experimental data allowed us to unambiguously identify proteasomal [63] and ribosomal [62] genes (Fig. 2a, **Experimental)**. Some of these genes were either not or falsely predicted when using a sequence-based approach. Of the annotated genes, 92% were confirmed by RNA reads. A total of 1148 *V. necatrix* genes could not be functionally annotated (Fig. 2a) using neither traditional nor structural annotation tools, as similarity hits were missing or of low confidence. Of those, hypothetical genes, that are conserved in several microsporidian species, were termed “uncharacterized”, whereas others that are only conserved within the order Nosematida were called “hypothetical”. RNA reads covered > 87% of the hypothetical genes suggesting that most hypothetical genes are expressed and not a result of an overestimated number of protein-coding regions predicted by BRAKER.

Further, we used the ribosome structure to unambiguously identify intron structures of seven ribosomal proteins (Supplementary Fig. 5) with the shortest intron counting 24 nucleotides. Alongside the microsporidian *Paranosema locustae*, *V. necatrix* belongs to the microsporidian species that maintain an operational splicing apparatus [25]. Additionally, the rRNA sequence, validated with the ribosome structure, allowed us to map the rDNA genes with high confidence (Fig. 2b). The pseudo-haplotypes contain 18 (pseudo-haplotype 1 and 3) or 20 (pseudo-haplotype 2 and 4) full rDNA loci, which are not clustering as repeats or localizing to subtelomeric regions as observed in *E. cuniculi* [64]. The rDNA loci are distributed among all chromosomes except for chromosomes 8 and 9. About half of the 12 to 14 copies of the 5 S gene localize to chromosome 6, while the additional copies are distributed on other chromosomes and generally closer to the chromosome ends (Fig. 2b).

To obtain a global view of the most abundant protein folds in *V. necatrix*, we performed an all-to-all Foldseek search and visualized structurally related proteins with a network graph (Fig. 2c). The most common protein fold in *V. necatrix* is represented by WD40 repeat domain-containing proteins followed by transposon-related proteins. While WD40 repeat domains are known as one of the most plentiful domain families in eukaryotes and are involved in protein-protein interactions [65, 66], the abundance of transposon-related elements in microsporidia can vary with the size of the genome. Overall, gene-sparse microsporidian genomes range from 12 to 50 Mb in size (Fig. 1a), and their non-coding regions are predominantly found to be transposable elements [67]. In our 15 Mb *V. necatrix* genome, we annotated around 109 retrotransposable elements that are involved in genetic mobility and genomic plasticity (Fig. 2c). In contrast, in the gene-dense genome of *E. cuniculi* (2.9 Mb), no such elements or RNA-dependent reverse transcriptases were identified, apart from the telomerase reverse transcriptase [8]. Additionally, we identified a Dicer-like protein (VNE69_01137) and an Argonaute protein (VNE69_01023), which belong to the RNAi machinery. A functional RNAi pathway correlates with a higher proportion of transposable elements and larger genome sizes [67] which might explain the high number of transposable elements found in *V. necatrix*.

Apart from eukaryotic conserved protein families (e.g., ABC transporters, kinases, AAA+ ATPases), *V. necatrix* harbors a large amount of Serine protease inhibitors (Serpins), RBL-like proteins (discussed below), and SWPs (Fig. 2c). To date among microsporidia, Serpins were exclusively found in Nosema and Vairimorpha [68], a genus infecting insects. One of the defense mechanisms of insects against pathogens is hemolymph melanization, which relies on the serine protease-mediated prophenoloxidase activation cascade. This process results in the inactivation of pathogens due to the deposition of melanin onto the invaders. Microsporidian Serpins were suggested to be secreted during host invasion to inhibit the prophenoloxidase activating proteinase, thereby interfering with the host’s innate immune response [21, 69]. This melanization pathway is conserved in Lepidoptera [70, 71], the host of *V. necatrix* [57], providing a potential reason for the enriched repertoire of Serpins we identified in this study. In fact, 0.81% of all *V. necatrix* genes encode Serpins, which is four times more than in *V. apis*, *V. ceranae* and *Nosema granulosis* and two-fold more than in *N. bombycis* as assessed by a UniProt search. Furthermore, the outermost layer of the mature microsporidian spore was shown to include many SWPs [72]. Since the spore wall is thought to be the first and most direct contact point with the environment and the host cell, the SWPs have potentially crucial roles in signaling, adherence, or enzymatic interactions [73]. Further studies are required to analyze the importance of these protein families for parasite adherence, invasion, and host immune evasion mechanisms.

### Enhancing automated annotations through structural similarity searches followed by manual curation

**Fig. 3:**
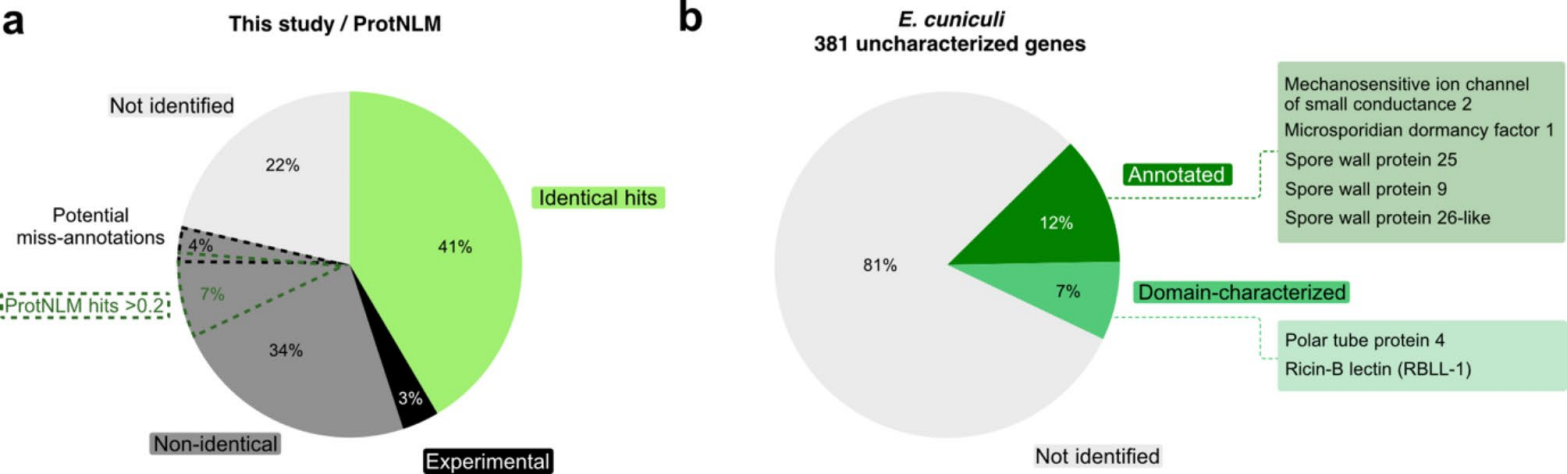
Complementation of structure and sequence-based functional annotation enriches the total number of matches and improves the annotation of microsporidia-specific genes. (**a**) To assess the annotation efficiency of our combined structure and sequence-based similarity approach, we counted the amount of identical (green), non-identical (dark grey), not identified (light grey) and experimentally determined (black) functional gene predictions between ANNOTEX and ProtNLM. Additionally, we display the relative number of potential miss-annotations (dark grey with black dashed line) predicted by ProtNLM and the percentage of ProtNLM gene function predictions with a model score above 0.2 (dark green dashed line) that we transferred to genes which our approach suggested to be uncharacterized or hypothetical. (**b**) Employing our approach, we functionally annotated 12% (dark green) and characterized the domain of 7% (green) of the 381 uncharacterized *E. cuniculi* proteins. RBLL-1, ricin B lectin-like 1

To benchmark our approach, we compared the final gene function annotations from our method to those from ProtNLM (Fig. 3a). In a second step, we employed our workflow on the uncharacterized genes from *E. cuniculi* retrieved from UniProt (accessed October 2022) [8], to test if we can improve the annotations that were recently updated with ProtNLM [74].

When comparing our ANNOTEX annotations with the assignments of ProtNLM, 42% of the gene function predictions had the same name or description, and 33% were different at first glance and were scrutinized below. Both ANNOTEX and ProtNLM failed to annotate 22% of all gene functions, while 3% could be assigned using solved protein structures [62, 63]. The 42% annotations with the same name or description, included cases where i.e., ProtNLM predicted a gene function while ANNOTEX identified a domain typically fulfilling this function, or vice versa. These consensus predictions reinforced the assigned gene function. Among the 33% different annotations, which account for 1009 genes, were 639 predicted gene functions made by us, for which ProtNLM provided low-confidence predictions with a model score below 0.2, an exclusion threshold used for UniProt annotations (https://www.uniprot.org/help/ProtNLM). For 229 out of 370 uncharacterized or hypothetical proteins according to ANNOTEX, ProtNLM predicted a domain description and/or gene function with a model score > 0.2. For these 229 proteins, we carried the predictions made by ProtNLM over to our functional annotation. Additionally, 14 microsporidia-specific gene functions were predicted with high confidence by our approach but were not recognized by ProtNLM. The remaining 126 (from 1009) seemingly different annotations required a closer look. Some proteins were assigned with a different (domain) function i.e., transposable elements (ANNOTEX: endonuclease vs. ProtNLM: integrase). However, the proteins in question might harbour both domains or fulfil both functions, whereas the prediction tools may only provide their preferred name. Thus, biochemical analysis would be necessary for confirmation. Further different annotations included non-informative predictions by ProtNLM such as DUF (domain of unknown function), phage protein, or WD40-repeat domain-containing proteins. For these cases, our manual curation step allowed us to visualize the proteins of interest and to find the best structural match, which increased the confidence in our functional prediction of the proteins. Further, up to 4% (121 genes) of all ProtNLM annotations include potential miss-annotations which are among the “non-identical” hits (Fig. 3a). Predictions like “Phage protein”, “Pine wood nematode protein”, “Plasmodium variant antigen protein Cir/Yir/Bir”, “Flagellar FliJ protein”, “Pilus assembly protein”, “Occlusion-derived virus envelope protein E66” seem to be incorrect annotations for microsporidia at least on the name-level. However, 61 of the 121 potentially miss-annotated gene functions are related to other obligate intracellular pathogens such as apicomplexans. The genes include surface and secretory proteins that aid in the parasitic lifestyle and are associated with the invasion into a host cell, formation of a parasitophorous vacuole, and replication. Since roughly one-third of these genes have a prediction model score above 0.2 in ProtNLM, it is likely that microsporidia share certain protein features with other intracellular parasites [75, 76]. However, instead of automatically carrying over the exact annotation i.e., “oocyst capsule protein”, we suggest annotating these as “oocyst capsule protein-like”.

We next tested our structural similarity approach on the 381 uncharacterized proteins from *E. cuniculi* (strain GB-M1) [8], for which the current functional prediction is sequence-based and was recently updated with ProtNLM annotations on UniProt [74]. By manually curating every protein, we could functionally annotate 46 proteins, and characterize domains in 26 proteins (Fig. 3b, Additional file 2). Our approach showed a clear advantage for microsporidia-specific genes that encode PTPs and SWPs and for proteins characterized via experimental structural analyses. We identified three microsporidia-specific SWPs (Q8SVI9, Spore wall protein 25; Q8SV25, Spore wall protein 9; Q8SVK8, Spore wall protein 26-like), two RBLs (Q8SUK2, ricin B lectin (Polar tube protein 4); Q8SUY7, ricin B lectin-like protein 1 (RBLL-1)) and the microsporidian dormancy factor 1, MDF1 (Q8SWQ4). Further, we annotated the gene coding for the mechanosensitive ion channel of small conductance 2 (Q8STV6), of which only a single copy exists in every microsporidian genome sequenced to date [11]. None of these assigned protein functions or domains were identified by ProtNLM, suggesting that structural similarity is an important complementary approach to predicting protein functions and characteristics in divergent organisms.

### For many divergent microsporidian proteins, structures are more conserved than sequences

**Fig. 4:**
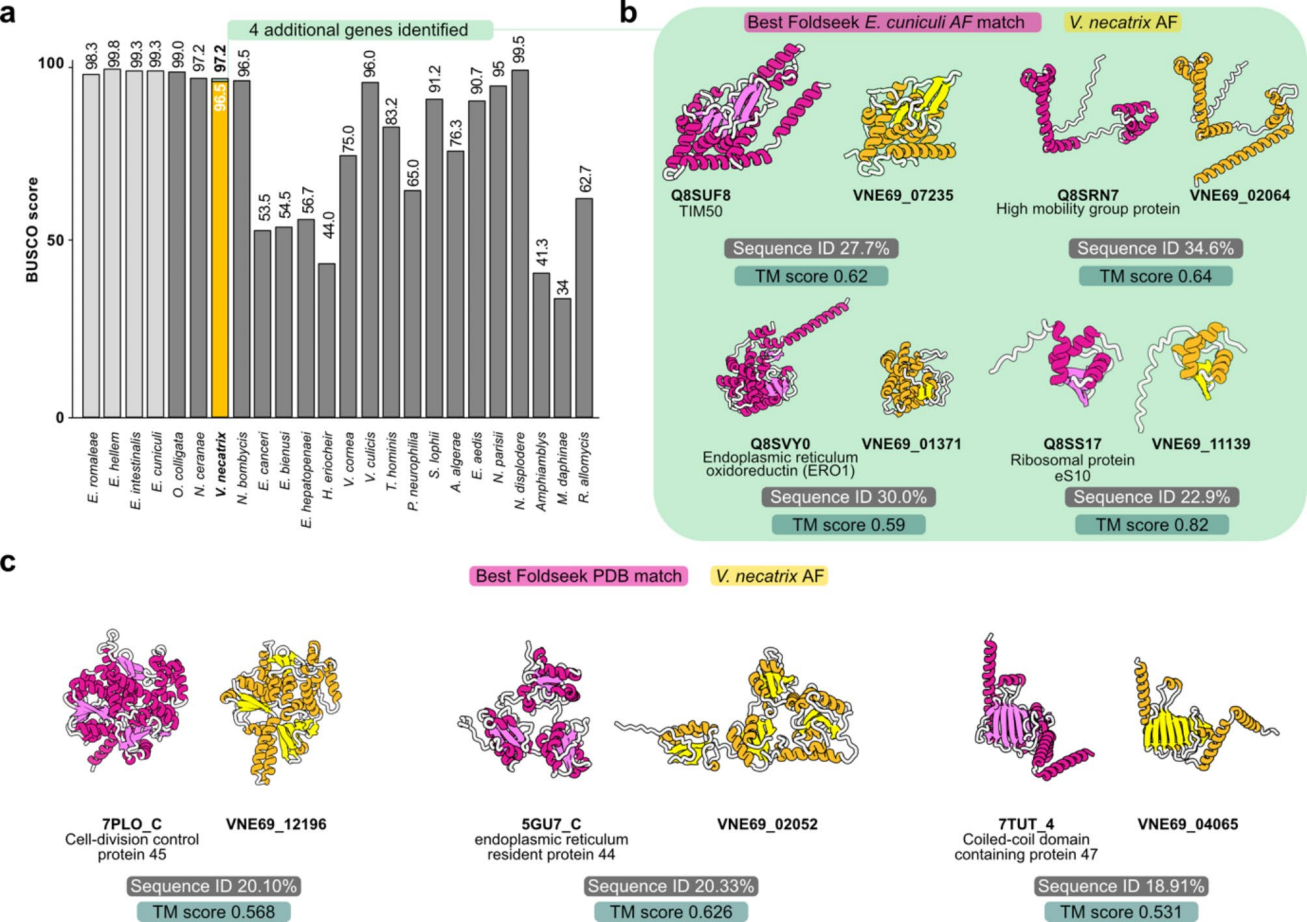
Examples of high-confidence structure-based hits for BUSCO genes, cell-division cycle and endoplasmic reticulum resident proteins. (**a**) BUSCO scores of a selection of microsporidian genomes compared to the score of *V. necatrix*. The genus Encephalitozoon is colored light grey. The *V. necatrix* BUSCO score bar is colored yellow with an extension in green representing the four additional genes identified using Foldseek. (**b**) AlphaFold structures of *E. cuniculi* (magenta) and *V. necatrix* (gold) proteins corresponding to the four microsporidia BUSCO genes. These four genes were exclusively identified via structural matching due to their low protein sequence identity. (**c**) Unambiguous identification of cell-division control protein 45, endoplasmic reticulum resident protein 44 and coiled-coil domain-containing protein 47 through structural similarity searches. Sequence-based searches lead to moderate-to-low-confidence hits comprising uncharacterized proteins, annotated protein domains or proteins with incorrect functional annotation. Sequence identity was calculated with ClustalW (v2.1), and TM scores were generated using TM-align (https://zhanggroup.org/TM-align/). TM score was normalized according to the length of the reference protein. Gold: Identified microsporidian proteins; magenta: Homologs; AF, AlphaFold; PDB, Protein Data Bank

Using structural similarity matching, we identified several proteins that were not identified previously through sequence similarity alone. For example, we attempted to retrieve the proteins corresponding to the eleven genes that were not identified during the BUSCO search, which is purely sequence-based (Table 1; Fig. 4a). Since large differences in the gene content can occur within higher taxonomic levels, it is necessary to use a specific BUSCO data set for the species of interest [77]. For microsporidia, the set of 600 reference genes to determine the BUSCO score stems from the Encephalitozoon genus (Fig. 4a). To identify the 11 missing genes, we folded the corresponding *E. cuniculi* proteins using ColabFold and performed structure-based matching with Foldseek to the *V. necatrix* proteins encoded by the predicted genes. With high confidence, four out of eleven missing genes were identified, increasing the BUSCO score slightly. The identified proteins displayed a high TM score but a low sequence similarity. The additionally matched proteins were the Endoplasmic reticulum membrane-associated oxidoreductin (ERO1), Mitochondrial import inner membrane translocase subunit TIM50, High-mobility group protein, and Ribosomal protein eS10 (Fig. 4a, and 4b). An additional protein (RING-type E3 ubiquitin transferase) that was identified had a low TM score, potentially due to disordered regions and high flexibility linkers. For the other six proteins, no clear best hit could be retrieved.

We further identified proteins involved in the cell-division cycle, membrane protein biogenesis and another endoplasmic reticulum resident protein using structural similarity searches. Blasting the sequence of these proteins gives a list of hits led by microsporidian proteins of more than 30% sequence identity, followed by other organisms, such as fungi, whose proteins show a sequence identity below 30%. As for microsporidian proteins, the origin and correctness of their functional annotation are sometimes vague and thus need further examination. For example, the two top Blast hits for VNE69_12196 are “Ribosomal protein l24e” (E-value = 2e^−^133) from *V. ceranae* (A0A0F9YQ74) and *Nosema ceranae* (C4V8F3). However, we annotated all ribosomal proteins of *V. necatrix* using the corresponding ribosome structure [62] which disagrees with this annotation. A Foldseek search on VNE69_12196 against the PDB100 database, using both algorithms 3Di/AA and TM-align, suggests “cell-division cycle protein 45” as high confidence structural match (Foldseek search E-value: 8.22e^− 10^ and TM score: 0.602) (Fig. 4c **left panel**). Additionally, a case where a structural similarity search provides a more contextual functional annotation, while Blast hits only contain a characterized domain, is VNE69_02052 (Fig. 4c **middle panel**). The most frequent sequence-based hits are “Thioredoxin domain-containing protein” which is correct but less informative compared to the high-confidence structural match “endoplasmic reticulum resident protein 44”. Lastly, structural similarity is a great tool for functional annotation when it comes to VNE69_04065 since a Blast search results in a list of uncharacterized and domain characterized proteins with ≤ 25% sequence identity. Using Foldseek however, we could annotate VNE69_04065 with high confidence (Foldseek search E-value: 1.91e^− 7^ and TM score: 0.620) as “coiled-coil domain-containing protein 47 (CCDC47)” (Fig. 4c **right panel**) which is part of the heterodimeric intramembrane chaperone complex (also PAT complex), that aids in membrane protein biogenesis in the endoplasmic reticulum [78, 79]. Through this functional annotation we were further able to identify the binding partner “Asterix” (VNE69_01152). A subsequent AlphaFold multimer prediction of the annotated CCDC47 and Asterix protein with a high confidence output model indicates that *V. necatrix* conserves the PAT complex to chaperone TMDs of membrane proteins and facilitate their biogenesis.

### Structure-based identification and classification of the expanded RBL family in microsporidia

Large expanded gene families in obligate intracellular pathogens are postulated to have an important role in host-pathogen interactions [80]. Among microsporidia, leucine-rich repeat-containing proteins are commonly found in the order Nematocida [81], while Serpins [68, 69, 81] and RBLs were shown to be abundant in Nosematida [30–32, 81]. RBL proteins belong to the β-trefoil fold lectins, a class of carbohydrate-binding proteins [82] that can aid in pathogen adherence to host-cells [83]. For example, 52 RBL proteins were identified in the silkworm pathogen *N. bombycis* [32] where they were shown to enhance spore adhesion and host-cell invasion [30]. However, the authors predict that 22 of these proteins lost their RBL domain due to extreme sequence divergence in microsporidia [32]. An alternate explanation might be that sequence-based methods are insufficient to identify the RBL domain or that previously, proteins were erroneously annotated as RBL domain-containing proteins [34, 84]. To test this hypothesis, and to unambiguously detect RBLs present in microsporidia we complemented the existing sequence-based search with our approach. For this, we focused on RBLs in Nosematida to demonstrate our structure-based workflow for finding homologs with low sequence identity.

**Fig. 5:**
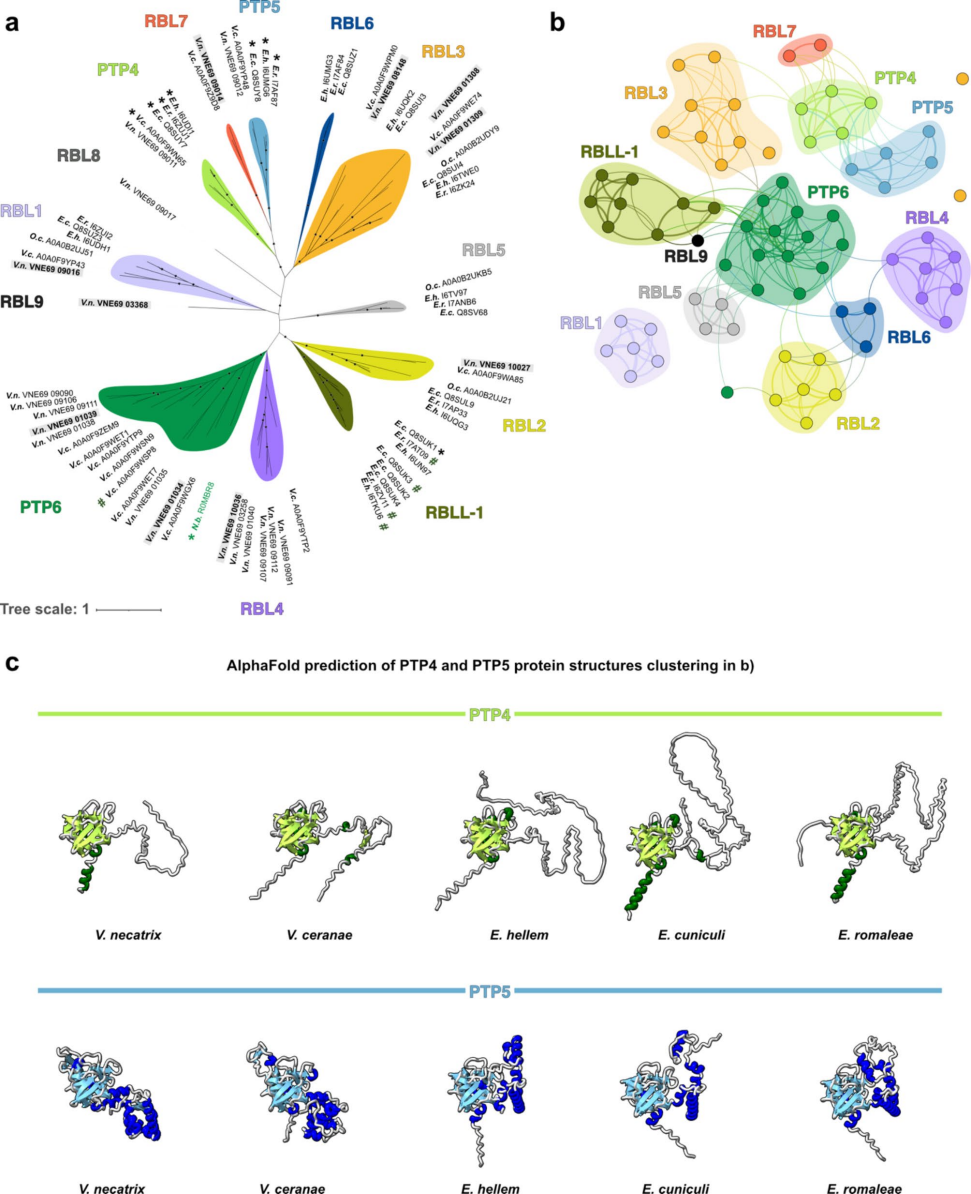
Structure-based identification and classification of the abundant RBL protein family. **a**) Cladogram of Nosematida RBLs named based on available experimental data (PTP4, PTP5, PTP6, RBLL-1) and otherwise termed RBL1 through RBL9. Branches marked with stars indicate a bootstrap value > 70. Protein IDs with asterisks indicate existing publications on the respective gene, hashtag marks indicate previously identified orthologs to NbPTP6 [86], and proteins in bold with a light grey background indicate the corresponding ten most highly expressed genes during germination. **b**) Structure-based network of RBL domain folds color-coded according to their clade in a). Each node represents one RBL domain, connecting lines indicate the degree of structural relatedness, and surrounding shapes in brighter shades mark structural clusters. Protein folds of all RBLs identified in a) were predicted with AlphaFold and RBL domains were clustered according to structural similarity based on their TM score using Gephi (v0.9.2) [91]. RBL8 was excluded as the AlphaFold prediction was of very low confidence. **c**) AlphaFold-predicted protein structures for the PTP4s and PTP5s comparing tertiary structures of the RBL domain between the two protein families and the microsporidian families. *E.c.*, *Encephalitozoon cuniculi*; *E.h.*, *Encephalitozoon hellem*; *E.r. Encephalitozoon romaleae*; *N.b.*, *Nosema bombycis; O.c.*, *Ordospora colligata*; *V.n.*, *Vairimorpha necatrix*; *V.c.*, *Vairimorpha ceranae*; RBL, ricin B lectin; RBLL, ricin B lectin-like; PTP, polar tube protein

We identified a total of 74 RBLs of which 22 were found in *V. necatrix* and several previously not identified in other species. We clustered them into 13 different RBL clades (Fig. 5a), of which four contain previously characterized proteins. These four include the PTP4, PTP5, PTP6, and RBLL-1 clades (Fig. 5a). Proteins from these clades all localize to different parts of the microsporidian PT [27, 85–87] or the spore wall [31]. PTP4 and PTP6 were shown to mediate host cell binding, while RBLL-1 from *E. cuniculi*, was shown to interact with the PT and spore wall [31]. The remaining nine uncharacterized RBL groups in our cladogram were termed RBL1 through 9. The clades RBL1 and RBL2, similar to PTP4 and PTP5, are conserved among microsporidia, as all species (except for *O. colligata* in the PTP4 and PTP5 clades [88]) are represented with one gene each. In contrast, members of the clades PTP6 and RBL4 were only identified in *V. ceranae* and *V. necatrix*. The RBLL-1 clade is represented exclusively by Encephalitozoon species, with *E. cuniculi* harboring the most RBL proteins (Q8SUK1 through 4). In the PTP4, PTP5, PTP6, RBL4, and RBLL-1 groups, almost all corresponding genes form clusters in the respective microsporidian genomes. *ptp4* and *ptp5* are always adjacent in all the genomes analyzed in this study, a finding previously reported for other microsporidian species [85]. Most of the *ptp6* genes are localized adjacent to *rbl4* genes in our *V. necatrix* genome (Additional file 1), and the four *rbll-1* genes in *E. cuniculi* form a gene cluster as well. These lineage-specific expansion of *rbl* genes in microsporidia could result from gene duplication events in response to the host immune system during infection and the subsequent evolutionary pressure on microsporidia to re-optimize host-cell attachment. Alternatively, their genomic closeness could indicate functional or physical interaction.

Next, to analyze the structural relationship among Nosematida RBLs, we generated a structure-based RBL-domain network using the TM score as a measure of structural similarity (Fig. 5b, Supplementary Fig. 6). We found that almost all RBL/β-trefoil domains corresponding to the RBL groups in Fig. 5a also cluster structurally, suggesting conserved function within the respective RBL clades and across species of the Nosematida order. Exceptions are the domain folds of one PTP6 (*V necatrix* VNE69_09111) and two RBL3 members (*V. necatrix* VNE6908_148 and *V. ceranae* A0A0F9WPM0). For these three RBL folds, we observed however that one of three β-trefoil subdomains is incomplete or missing, or the prediction for this subdomain is of low confidence. This suggests that the structural clustering is inaccurate and not reliable for these three RBL members. Regarding structural relations between clusters, most RBL domains show structural similarity to the PTP6 clade members which form a center in this network. Only the RBL domain clusters from the clades RBL7, PTP4, PTP5 and RBL1 have no direct connection to the PTP6 domain family. In fact, the RBL1 domain folds have no connection to any other RBL class but form a joint cluster with all clade members identified in Fig. 5a (indicating their TM score with all other RBL domains is < 0.7). Taking a closer look at the 3D protein model, the RBL1 β-trefoil domain harbors an additional (well predicted) β-sheet pair in a loop region, which is absent at this position in all other RBL domain folds. Since RBL1 is present in every Nosematida species analyzed here, it is possible that the additional β-sheet pair is beneficial for carbohydrate interaction (stabilization). In *V. necatrix*, *rbl1* is among the ten most highly expressed *rbl* genes during germination (Additional file 1) suggesting that it is involved in microsporidian host-cell invasion.

Since both PTP4 and PTP5 are unique to microsporidia and form part of their infection apparatus, we were interested in their structural similarities and differences, based on the AlphaFold predictions (Fig. 5c). The structural network indicates that all PTP4 RBL-domains share a high structural similarity, while PTP5 from *V. necatrix* and *V. ceranae* seem structurally less related to the Encephalitozoon homologs, possibly due to an additional β-sheet pair, incorporated in the RBL domain (different position than in RBL1) (Fig. 5c, **lower panel**). This could be a host specific trait and beneficial for *V. necatrix* and *V. ceranae* infection of moth larvae and honeybees, respectively. Other than that, the RBL-domain folds of PTP4 and PTP5 are nearly identical, suggesting a high conservation of the 3D structure. This high conservation is essential, as for example *E. hellem* PTP4, localized at the PT tip, where the infectious cell content is transferred from the PT into the host cell, was shown to interact with host-cells during *E. hellem* host-cell invasion [27].

Structural similarity searches allowed us to identify new members of the large RBL protein family in Nosematida. We also showed that PTP4-6 are members of the RBL family, which may contain additional, yet uncharacterized proteins that form part of the unique microsporidian infection apparatus. A recent study identified multiple RBL proteins as interaction partners of *V. necatrix* PTP3 [89], one of the main components of the PT [90]. Our findings of the close relationship between PTP4-6 and RBL proteins, and the interaction of various RBL proteins with the microsporidian PT indicate an important role of RBLs in microsporidian host-invasion and incentivize further experimental research on this large protein family in Nosematida.

## Conclusion

The functional annotation of proteins is a critical step for understanding the biology of organisms. Even though automated annotations are essential to whole genome/proteome projects, they traditionally rely on sequence similarity, orthology searches, and protein name predictions based on the amino acid sequence. This poses three major problems: First, sequence similarity searches can fail to result in significant matches if the sequence is too divergent from the ones present in databases. This is often the case when analyzing understudied species like microsporidia or newly emerging pathogens. Second, up to date, low sequence identity blast hits against *S. cerevisiae* and other model organisms led to functional annotation of microsporidian genes that are neither in accordance with the structural hits identified by Foldseek nor with the ribosomal and proteasomal genes revealed through structural studies [62, 63]. Third, any previous annotation error is likely to be propagated across species. Thus, for divergent species with low sequence identity like microsporidia, sequence-based annotations are not sufficient. However, since the structure and the biological role of a protein are connected, protein function can be inferred using structural similarity searches. We developed a functional annotation workflow that allowed us to manually curate sequence and structure-based matches and to select the best hit based on sequence similarity and TM score. We used this annotation workflow on our newly sequenced, high-quality genome of *V. necatrix*, a microsporidian species poorly characterized up to this point.

The implementation of structural similarity searches and the manual curation step, that ANNOTEX offers, allows us to identify potential miss-annotations and may thus prevent their automatic transfer in the future. Further, it is possible to filter out proteins that are exclusively present in invertebrates and are most likely contaminants. Our pipeline, complemented with ProtNLM, allowed us to functionally annotate 1932 out of 3080 predicted genes (2971 genes of *V. necatrix* and 109 TEs), including 319 hits identified with ANNOTEX that could not be identified using traditional sequence-based approaches only. The complementary information from sequence and structure further allowed us to characterize 19% (72 proteins out of 381) of the *E. cuniculi* proteins or protein domains that were previously annotated as “hypothetical” or “uncharacterized”. Further, using structural similarity searches, we have identified previously unknown RBL family members in the order Nosematida and shown that PTP4, PTP5, and PTP6 are part of the RBL family. Structural information gives a first hint of the putative function of a protein, its structural appearance, and potential interaction partners and may thus provide guidelines for experimental analyses and biochemical verification.

Thorough analyses of microsporidian genomes are essential to identify and functionally characterize species-specific proteins, which can provide novel drug targets to fight microsporidiosis in humans as well as environmentally and economically important animals. The identification of potential drug targets requires reliable tools to accurately identify and characterize divergent genes in microsporidia. Our approach improves the quality and quantity of functional genome annotation of a divergent organism and presents the first high-quality genome and annotation of the microsporidian *V. necatrix*.

Even though our approach requires a manual curation step, structural similarity tools for protein annotation are an important complement to traditional sequence annotation tools and aid in overcoming annotation challenges with divergence and long evolutionary distance. We expect structural similarity searches to become even more powerful as additional reference structures become available and as structural prediction tools continue to improve. ANNOTEX is a valuable tool for the accurate functional annotation and curation of genomes obtained from highly divergent, non-model organisms.

## Methods

### ***V. necatrix*** genomic DNA extraction

*V. necatrix* spores were propagated in the fourth and fifth instar larvae of *Helicoverpa zea* (corn earworm). The larvae were homogenized in Fisher 50 mL closed Tissue Grinder System tubes in water, filtered through a double layer of cheesecloth, and further filtered through 100 and 40 μm Biologix centrifugal filters before storage at − 80 ˚C until further use. For genomic DNA extraction, *V. necatrix* spores were thawed, purified over 100% Percoll, and washed three times with sterile MilliQ water before the spore homogeneity was assessed by light microscopy. 12 mg of highly pure spores were germinated using the alkaline priming method [92]. Spores were resuspended in 200 μl 0.1 M KOH for 20 min at 22 °C, pelleted via centrifugation at 2000 x g for 2 min, and resuspended in 100 μl germination buffer (0.17 M KCl, 1 mM Tris-HCl pH 8.0, 10 mM EDTA). A germination rate of approximately 80% was observed by light microscopy. To extract genomic DNA from the germinated spores, the Monarch® Genomic DNA Purification Kit (NEB, Cat# T3010) was used (10 μl Proteinase K, 3 μl RNase). Genomic DNA was eluted twice with 80 μl sterile MilliQ water. DNA quantification and qualification were assessed by Nanodrop and Qubit. Additional DNA quality assessments included electrophoresis on a 0.8% agarose gel stained with ethidium bromide and PCR amplification of a control gene.

### Sequencing and assembly

The extracted *V. necatrix* genomic DNA was sent to the National Genomics Infrastructure (NGI) Uppsala Genome Center (Science for Life Laboratory, Uppsala, Sweden) for PacBio *de novo* sequencing. To prepare the sequencing library for PacBio sequencing, 2 μg of genomic DNA were sheared on a Megaruptor3 instrument (Diagenode, Seraing, Belgium) to a fragment size of about 18 kb. The SMRTbell library was prepared according to PacBio’s Procedure & Checklist – Preparing HiFi Libraries from low DNA input using SMRTbell Express Template Prep Kit 2.0 (Pacific Biosciences, Menlo Park, CA, USA). The SMRTbells were sequenced on a Sequel II instrument, using the Sequel II sequencing plate 2.0, binding kit 2.2 on one Sequel® II SMRT® Cell 8 M, with a movie time of 30 h and a pre-extension time of 2 h.

The sequencing resulted in 2’053’200 HiFi-reads (mean QV = 34) with a total of 28 gigabases and an N50 read length of 13.7 kb. The dataset was split into 14 equal-sized read sets. Read sets were assembled using hifiasm (v0.16.1). The resulting assemblies were split into 4 pseudo-haplotypes and the sequence identity assessed by MUMmer/dnadiff (v3.23) [52]. Contig ends were inspected for all assemblies to identify the telomeric repeat units. Telomere-to-telomere contigs were selected for each chromosome and verified using telomeric-identifier (v0.2.41). The final assembly was then polished using Flye (v2.8.3).

### Sample preparation for RNA seq

20 mg of highly germination-competent *V. necatrix* spores (> 80% germination efficiency), stored at − 80 ˚C, were thawed and cleaned by centrifugation through a 50% Percoll cushion. Subsequently, three MilliQ water washes were performed to remove Percoll remnants. Germination of cleaned spores was performed by alkaline priming of the spores in 200 μl of KOH followed by adding germination buffer (0.17 M KCl, 1 mM Tris-HCl pH 8.0, 10 mM EDTA). Gemination events were confirmed by light microscopy followed by the immediate addition of 300 μl of Ex-Cell 420 medium supplemented with 1 mM ATP. The sample was immediately added to an equal volume of Trizol reagent (Invitrogen Cat no. 15,596,026) and further supplemented with $${\raise0.7ex\hbox{$1$} \!\mathord{\left/{\vphantom {1 3}}\right.\kern-\nulldelimiterspace}\!\lower0.7ex\hbox{$3$}}$$ volume of zirconium beads. Samples were vortexed for 1 min and incubated on ice for 1 min. This step was repeated two more times. Samples were spun down at 20,000 x g for 10 min at 4 °C followed by withdrawal of the aqueous layer and two subsequent extractions of the aqueous layer with chloroform. Overnight RNA precipitation was done with 2.2 volumes of ice-cold 96% ethanol, 1/10 volume of 3 M sodium acetate (pH 5.2), and 1 μl of Glycol blue co-precipitant. The next day, RNA precipitates were pelleted by centrifugation and washed twice with ice-cold 75% ethanol. The pellet was dissolved in 20 μl of nuclease-free water and treated with RNase-free DNase 1 (Invitrogen EN0521). As control and confirmation, the RNA sample was run on a 2% agarose gel.

### RNA library preparation and NovaSeq sequencing

RNA samples were quantified using Qubit 4.0 Fluorometer (Invitrogen, Carlsbad, CA, USA), and RNA integrity was checked with an RNA Kit on an Agilent 5300 Fragment Analyzer (Agilent Technologies, Palo Alto, CA, USA). RNA sequencing libraries were prepared using the NEBNext Ultra RNA Library Prep Kit for Illumina following the manufacturer’s instructions (NEB, Ipswich, MA, USA). Briefly, mRNAs were first enriched with Oligo(dT) beads. Enriched mRNAs were fragmented for 15 minutes at 94°C. First-strand and second-strand cDNAs were subsequently synthesized. cDNA fragments were end-repaired and adenylated at 3’ ends, and universal adapters were ligated to cDNA fragments, followed by index addition and library enrichment via limited-cycle PCR. Sequencing libraries were validated using the NGS Kit on the Agilent 5300 Fragment Analyzer (Agilent Technologies, Palo Alto, CA, USA), and quantified with the Qubit 4.0 Fluorometer (Invitrogen, Carlsbad, CA, USA).

### RNA seq data quality assessment

RNA seq data was received as fastq reads. Quality was checked with FastQC (v0.11.9) and sequences were subsequently subjected to trimming using Trimmomatic (v0.33) to remove adapter contaminations and trim low quality bases using the option “ILLUMINACLIP:TruSeq3-PE.fa:2:30:10:2:keepBothReads LEADING:3 TRAILING:3 MINLEN:120”. The trimmed reads were then aligned to the predicted genes of pseudo-haplotype 1 with STAR (v2.7.10). FeatureCounts (part of Subread v2.0.3) was then used to count the number of reads, normalized by gene length and the resulting counts were plotted in a non-stacked bar plot in log10 bins (Supplementary Fig. 7a). Of all aligned reads, 79.53% were uniquely mapped reads, 4.97% of reads mapped to multiple loci, 6.19% of reads mapped to too many loci, and 9.29% of reads were unmapped. Unmapped reads could be poor-quality reads, missed genes in the original gene prediction, or contamination from the host.

### Gene prediction and annotation

Prior to gene prediction, potential transposable elements (TE) were identified using RepeatModeler (v2.0.3), a de novo transposable element identification and modeling package. Using default parameters, a database of TE families was built. Next, RepeatMasker (v4.1.0) was used to softmask the genome followed by gene prediction with ProtHint and Augustus via the BRAKER (v2.1.6) pipeline. The quality of the predicted genes was assessed using BUSCO (v5.4.3) against the microsporidia_odb10 dataset.

### Generating a database for the functional annotation with our ChimeraX annotator plugin ANNOTEX

For the functional annotation, a database was generated to retrieve the best sequence and structure-based matches for each input sequence. The sequence-based search was done using Diamond (v2.1.8) with the ultra-sensitive option against the non-redundant NCBI database. The eggNOG (v2.1.9) mapper was used with non-default parameters (Percentage identity: 15%, Minimum hit bit-score: 40) and allowed for functional annotation based on orthology predictions which is considered more precise than traditional homology searches. For structural matches we folded the *V. necatrix* proteome and the hypothetical proteins of *E. cuniculi* using ColabFold (v1.5.2) with default parameters. Next, we used each individual predicted 3D structure as input for Foldseek (v5-53465f0) searches employing the alignment type 3Di + AA Gotoh-Smith-Waterman (local, default) and ran it against three different databases: (1) PDB, (2) AlphaFold database from the 20 first annotated model organisms (accession date: 07-15-2022), one representative of each microsporidian clade (Fig. 1a), and (3) SwissProt AlphaFold. Additionally, for the *E. cuniculi* proteins, individual, well-predicted protein domains were automatically separated using the Predicted Aligned Error (PAE) [44] and subjected to the TM-align algorithm in Foldseek. As a measure of confidence, the E-value is displayed for all Diamond and eggNOG searches, while the significance of the Foldseek searches varies with the alignment type: The bit score assesses 3Di + AA Gotoh-Smith-Waterman search results and the TM score (global score) represents the confidence of TM-align searches. Further, to predict the overall 3D structure and the presence of a SP or TMD for each analyzed protein, the Deep Transmembrane Helix Hidden Markov Model (DeepTMHMM) (v1.0.20) software [93] was used.

To combine and display the generated information and similarity matches for each *V. necatrix* input sequence, we developed a ChimeraX annotator plugin, that we named ANNOTEX (Supplementary Fig. 3). It retrieves a list of all predicted *V. necatrix* protein 3D structures, shows the eggNOG annotation in the user interface (Supplementary Fig. 3b), and presents a list of structural matches and sequence-based hits, respectively, along with corresponding confidence values. Further, the proteins corresponding to structural hits can be superimposed with the *V. necatrix* protein of interest, allowing for visual inspection of the structure match. Additionally, the overview of all structural and sequence hits per protein allows for manual curation and functional annotation according to the best match.

### Analysis of false positive gene prediction of non-annotated genes

To estimate how many of the predicted hypothetical genes might be false positive genes, we compared the RNA sequencing reads between annotated and non-annotated genes (Supplementary Fig. 7a). More than 87% of the hypothetical genes are covered by RNA reads, which is close to the 92% coverage of the successfully annotated genes and suggests that most hypothetical genes are present. The hypothetical genes could either encode yet unknown proteins or are the result of an overestimated number of protein-coding regions predicted by BRAKER (v2.1.6). However, more than 550 of these genes have mRNA sequence reads over 200 (Supplementary Fig. 7a). In addition, we searched for the presence of CCC-like or GGG-like motifs 30 bp upstream from the start codon. The presence of these motifs was proposed to significantly improve the microsporidian genome annotation [94, 95]. For the *V. ceranae* and *E. bieneusi* genomes which do not display the CCC-like or GGG-like motifs, an AT content of > 80% 30 bp upstream of the translation initiation site was considered instead as a criterion to solidify start codons for these two species [94, 96]. In the *V. necatrix* genome, we identified a CCC or GGG motif in 77% of the genes encoding functionally characterized proteins, in 75% of the genes coding for hypothetical proteins, and in 54% of the predicted transposons (Supplementary Fig. 7b). The AT ratio between genes encoding predicted and hypothetical proteins was similar but lower in predicted transposons. Based on these results, it is likely that very few, if any, genes are false positives [94, 96]. Further, a significantly higher number of proteins with an SP and TMD is predicted among the hypothetical compared to the classified proteins (Supplementary Fig. 7c). Since both SPs and TMDs seem to be key features of host-exposed proteins [81], this abundance suggests that many of the hypothetical proteins belong to the group of exported proteins. Host-exposed proteins have been found to evolve faster than the remainder of the proteome, presumably because these proteins are under pressure from the host immune system. In Nematocida, it was shown that host-exposed proteins evolve rapidly and are most often lineage-specific [81]. Most of these proteins are thus hypotheticals and present a low evolutionary traceability which hinders further annotation efforts.

### Benchmarking

Shortly after we completed the functional genome annotation, the automated annotation tool ProtNLM (v2022_04) was published and represented the new standard for sequence-based annotation, replacing eggNOG. Therefore, we decided to benchmark our approach, and we manually compared the final gene function annotations that we generated with ANNOTEX for the *V. necatrix* genome and the *E. cuniculi* (strain GB-M1) uncharacterized proteins from UniProt [8] to the results from ProtNLM. We distinguished between identical annotations, different annotations, not-identified annotations, and experimentally determined gene functions which are based on published studies. The identical annotations also include cases where either tool, ANNOTEX or ProtNLM, predicted a gene function and the respective other tool predicted only a protein domain that is typically involved in this gene function. Differing predictions also include a subsection of potential miss-annotations made by ProtNLM. Not identified gene functions comprise hypothetical proteins, uncharacterized proteins, and DUF domain-containing proteins, independently of whether a feature like TMD or SP is listed. Additionally, for the *E. cuniculi* uncharacterized gene set, we differentiated between the characterized protein and the characterized protein domain. The number of proteins in each category was counted and displayed in a pie chart to visualize the performance of our annotation approach.

### RBL identification, analysis, and visualization

To identify RBL proteins in the order Nosematida using structural homology, characterized RBL domain-containing proteins, such as PTP4 and PTP5, were identified in *V. necatrix* and *E. cuniculi.* The corresponding AlphaFold models were extracted from the annotator database and large disordered regions were trimmed to retain only the well-predicted ricin-type β-trefoil lectin domain. This domain served as a template for structural homology searches in Foldseek using the TM-align algorithm. Among the homology matches were mannosyl transferases, which typically contain a functional RBL domain (i.e., VNE69_06039 and VNE69_12061) and were thus removed. HMMER profiles (v3.3.2) (http://hmmer.org) [97] were generated to detect RBL proteins that were potentially overlooked by the structural search.

Next, the sequences of all identified RBL domain-containing proteins were aligned with MUSCLE (5.1) [98] and trimmed with trimAl (v1.4.1) [99], a tool for the automated removal of spurious sequences and poorly aligned regions from a multiple sequence alignment. The remaining sequences were used to build a cladogram with IQ-TREE (v2.0.3) [100, 101] with 1000 bootstrap replicates and the MFP option for choice of substitution model.

To generate a structural network graph based on the high-confidence AlphaFold models for the RBL domains (Supplementary Figs. 4 and 6), the visualization software Gephi (v0.9.2) [91] was used according to the user guidelines. Briefly, the required nodes and edges data sheets were generated for which the squared TM score served as edge weight. Data was imported into Gephi, the graph type was set to undirected, statistic tools In/Out Degree, Network Diameter, Graph Density, Modularity, and Average Clustering were run using default settings, and ForceAtlas 2 was chosen as layout. To reduce clutter, we displayed only the five closest structural relations (five edges) of each RBL domain and only those that have a TM score > 0.7.

### Electronic supplementary material

Below is the link to the electronic supplementary material.

**Additional file 1: Annotation table** ***V. necatrix.*** Table “suppl_tbl_1_genome_annotations” contains all locus tags and the resulting structure-based annotations. Further, the ProtNLM results are included and the differences to our annotation are indicated (x: different annotations, y: non-identified, z: potential misannotations, e: experimental structure). In addition, the spore-0hr RNA sequence counts are listed per gene. Table “suppl_tbl_1_InterPro” contains all locus tags, the ANNOTEX annotations and all Interproscan (5.65-97.0-64-bit) results. Interproscan was executed with standard parameters and all available analyses except those that use licensed code

**Additional file 2: Annotation Table** ***E. cuniculi.*** List of 381 uncharacterized proteins from *E. cuniculi* and the updated annotation using our approach

**Additional file 3: Supplementary Figure 1.** Identification of telomers. **Supplementary Figure 2.** Complementary annotation pipeline from genome to function. **Supplementary Figure 3.** ANNOTEX overview. **Supplementary Figure 4.** AlphaFold2 pLDDT scores and structural prediction quality overall, of the final annotation and for the RBL protein regions. **Supplementary Figure 5.** Intron containing ribosomal proteins. **Supplementary Figure 6.** Structural network of ricin B lectins in Nosematida shown with organism and gene IDs. **Supplementary Figure 7.** RNA sequencing reads of annotated and unannotated genes, and protein features of hypothetical, uncharacterized, and classified proteins. **Supplementary Data File 1.** Data file with the ChimeraX plugin “ANNOTEX”, the used annotation database and all predicted structures

## Data Availability

The raw PacBio data and the final *V. necatrix* assembly including annotation information were deposited at NCBI under BioProject accession ID PRJNA909071 and BioSample SAMN32066506. Raw transcriptomics data were deposited under PRJNA909071. The ChimeraX plugin ANNOTEX and the databases built for annotation of the *V. necatrix* and the *E. cuniculi* genes can be accessed on Zenodo under 10.5281/zenodo.7974739 (10.5281/zenodo.7974739).
