# Supplementary material for: Functional annotation of a divergent genome using sequence and structure-based similarity
Source: BMC Genomics. 2024 Jan 2;25:6. doi: 10.1186/s12864-023-09924-y (PMC10759460; doi:10.1186/s12864-023-09924-y)
Supplement: Supplementary file 3 — Additional file 3: Supplementary Figure 1. Identification of telomers. Supplementary Figure 2. Complementary annotation pipeline from genome to function. Supplementary Figure 3. ANNOTEX overview. Supplementary Figure 4. AlphaFold2 pLDDT scores and structural prediction quality overall, of the final annotation and for the RBL protein regions. Supplementary Figure 5. Intron containing ribosomal proteins. Supplementary Figure 6. Structural network of ricin B lectins in Nosematida shown with organism and gene IDs. Supplementary Figure 7. RNA sequencing reads of annotated and unannotated genes, and protein features of hypothetical, uncharacterized, and classified proteins. Supplementary Data File 1. Data file with the ChimeraX plugin “ANNOTEX”, the used annotation database and all predicted structures [file 12864_2023_9924_MOESM3_ESM.pdf]

Supplementary Information for

Functional annotation of a divergent genome using sequence and structure-based similarity.

Dennis Svedberg[1,2,#], Rahel R. Winiger[1,#], Alexandra Berg[1,2,#], Himanshu Sharma[1,2], Christian Tellgren-Roth[3], Bettina A. Debrunner-Vossbrinck[4], Charles R. Vossbrinck[5], Jonas Barandun[1,*]

[1] Department of Molecular Biology, The Laboratory for Molecular Infection Medicine Sweden (MIMS), Umeå Centre for Microbial Research (UCMR), Science for Life Laboratory, Umeå University, 90187 Umeå, Sweden.

[2] Department of Medical Biochemistry and Biophysics, Umeå University, 90736 Umeå, Sweden.

[3] Science for Life Laboratory, Department of Immunology, Genetics and Pathology, Uppsala University, Uppsala, Sweden.

[4] Department of Math/Science, Gateway Community College, 20 Church Street, New Haven, Connecticut 06510, United States of America.

[5] Department of Environmental Science, Connecticut Agricultural Experiment Station, New Haven, Connecticut 06504, United States of America.

[#] These authors contributed equally to this work.

[*] Corresponding author: jonas.barandun@umu.se (J.B.)

Supplementary Figure 1. Identification of telomers.

Supplementary Figure 2. Complementary annotation pipeline from genome to function.

Supplementary Figure 3. ANNOTEX overview.

Supplementary Figure 4. AlphaFold2 pLDDT scores and structural prediction quality overall, of the final annotation and for the RBL protein regions.

Supplementary Figure 5. Intron containing ribosomal proteins.

Supplementary Figure 6. Structural network of ricin B lectins in Nosematida shown with organism and gene IDs.

Supplementary Figure 7. RNA sequencing reads of annotated and unannotated genes, and protein features of hypothetical, uncharacterized, and classified proteins.

Supplementary Data File 1. Data file with the ChimeraX plugin "ANNOTEX", the used annotation database and all predicted structures. (https://doi.org/10.5281/zenodo.7974739) V_necatrix_alphafold.zip (Alphafold models and associated files for all *V. necatrix* proteins), chimerax_annotater_plugin.zip (ChimeraX plugin install file), v_necatrix_annotation_data.zip (annotation data used in ANNOTEX and generated as described in the methods section; https://github.com/Barandun-Lab/ANNOTEX)

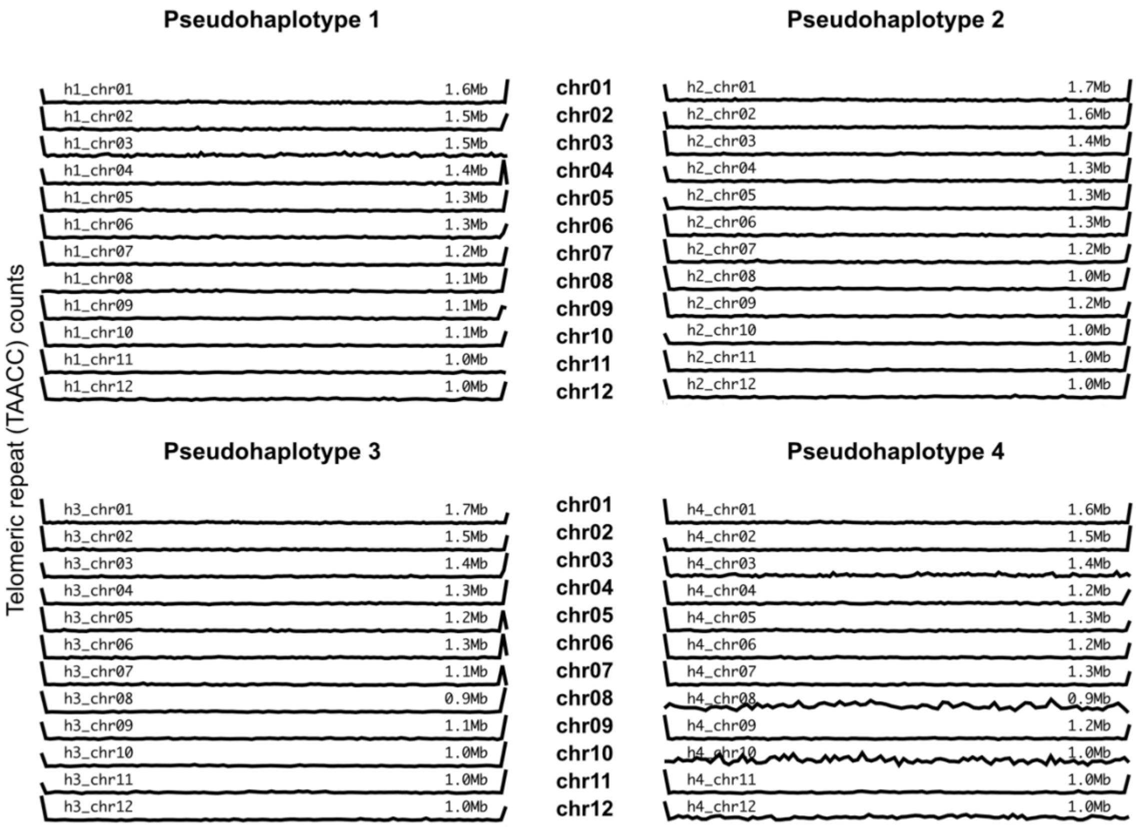

Supplementary Figure 1. Identification of telomeres. Output plots of telomere-identifier (v0.2.41) (https://github.com/tolkit/telomeric-identifier) for all chromosomes of the four pseudo-haplotypes showing telomeric regions on 42 of the 48 contig ends, confirming two complete telomere-to-telomere pseudo-haplotypes.

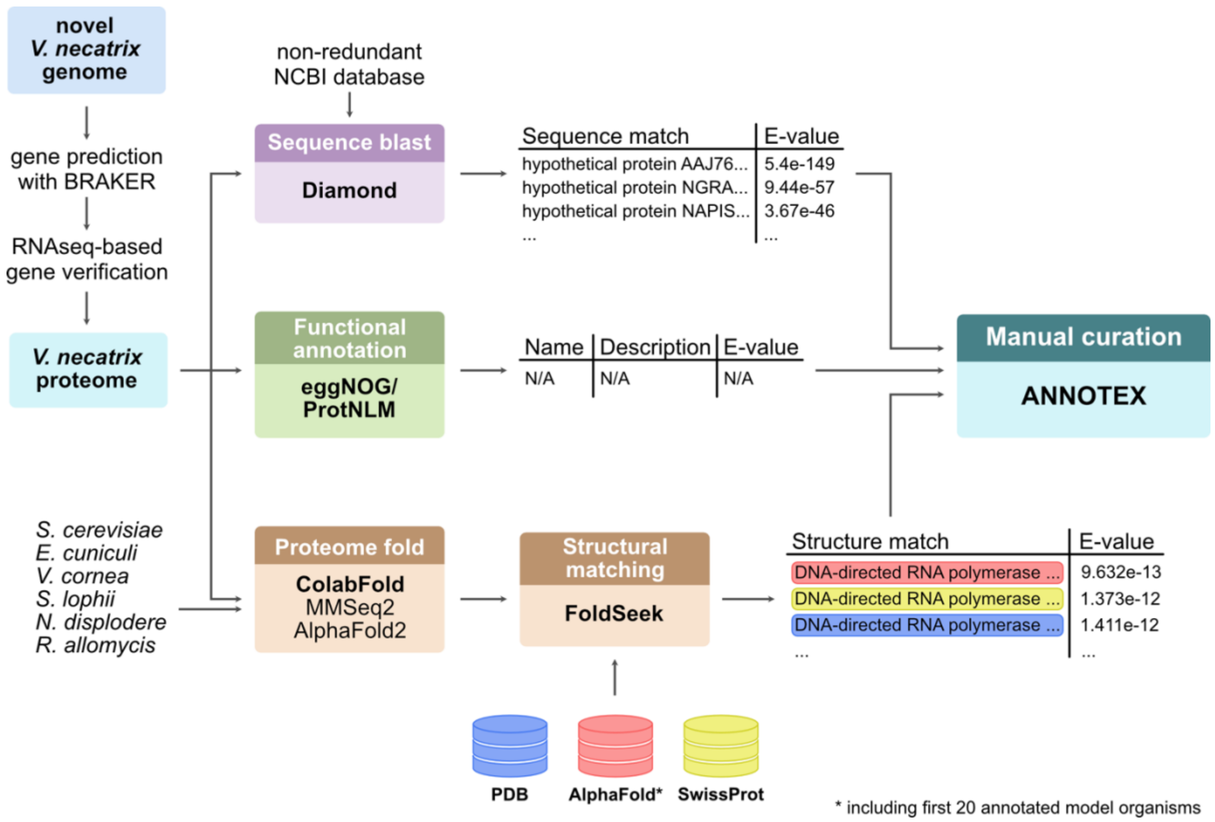

Supplementary Figure 2. Complementary annotation pipeline from genome to function. We predicted genes in our novel *V. necatrix* genome using BRAKER and verified the protein-coding regions with our transcriptomic data. The corresponding *V. necatrix* proteome served as basis for functional annotation using a combination of three different approaches: Sequence-based annotation with Diamond, functional annotation via domain homology using eggNOG and via natural language processing based on the amino acid sequence operated by ProtNLM, and structural similarity searches employing ColabFold and Foldseek. For the structural similarity search, we used ColabFold to fold the proteomes of *V. necatrix*, *S. cerevisiae* as representative model organisms, and five microsporidian species, each representing a clade. Among the folded proteomes, we searched for structural matches to the *V. necatrix* proteins and further used the databases PDB, AlphaFold/Proteome (accessed July 2022, only 20 folded proteomes of model organisms were available) and AlphaFold/SwissProt. The functional prediction matches and the corresponding E-value, bit score, or TM score of all three approaches were visually combined in ANNOTEX allowing us to find the best matches and manually curate the functional annotations.

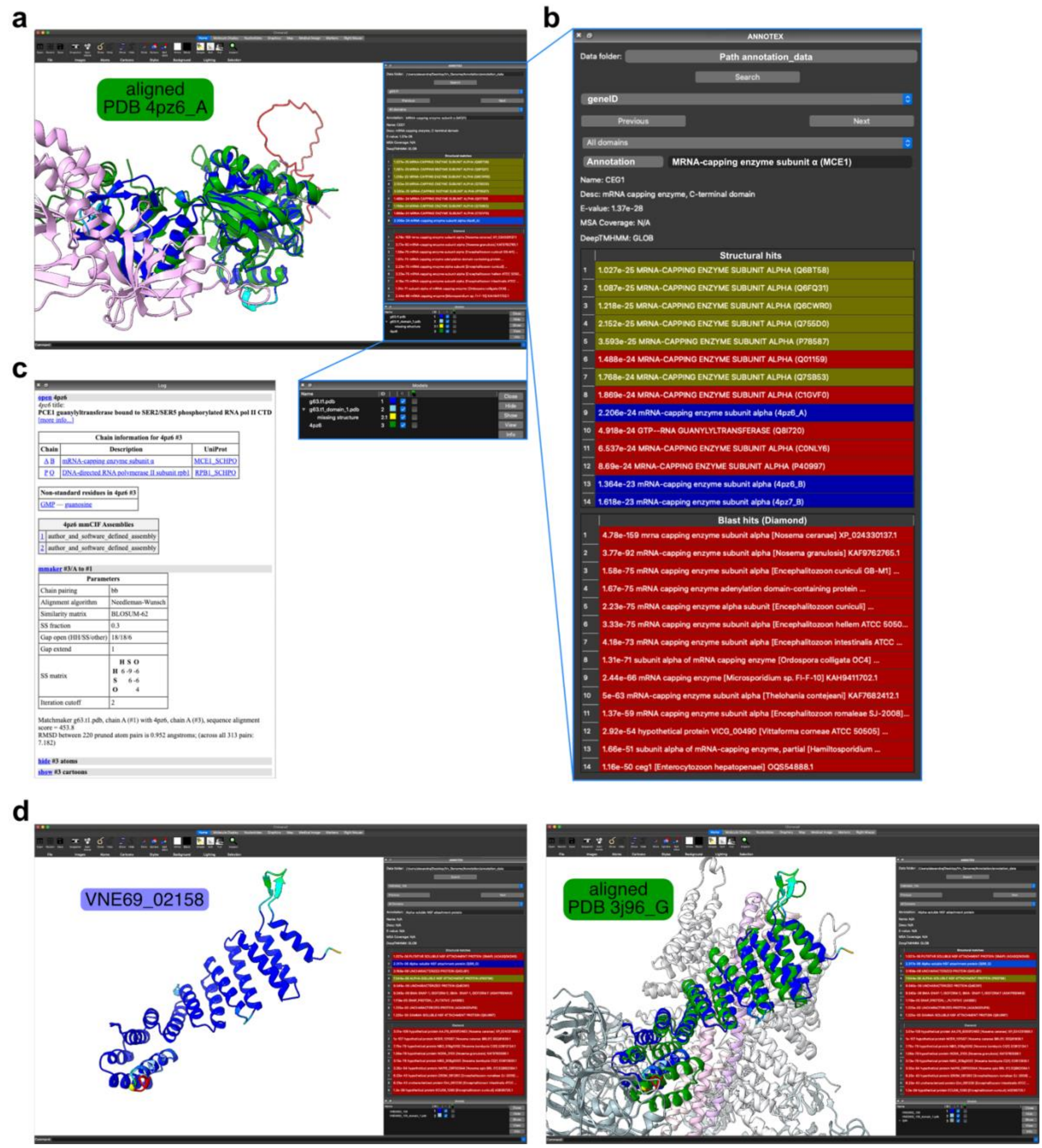

Supplementary Figure 3. ANNOTEX overview. (**a**) ChimeraX window and plugin showing the AlphaFold-predicted protein encoded by gene 63.t1 (now renamed VNE69_03064) and superimposed with a structural match (PDBid: 4pz6_A) in the 3D viewer. Lower panel: Zoom of Model window listing structures, domains, and missing structures displayed in the model viewer. (**b)** Our ChimeraX plugin "ANNOTEX" consists of a tool window docked to the right, from top to bottom: "Data folder" path search tool, gene ID bar, domain selection bar, potential eggNOG annotation with protein name, description, and E-value, MSA coverage, DeepTMHMM information, a list of structural matches from databases PDB, AlphaFold microsporidian proteomes, and AlphaFold SwissProt, followed by Diamond sequence blast hits. (**c**) ChimeraX Log console showing name, chain information, and parameters of the selected PDB structure. (**d**) ANNOTEX presenting the AlphaFold protein structure encoded by gene VNE69_02158 in rainbow colors (left panel) and superimposition with a structural match (PDBid: 3j96_G) in green (right panel).

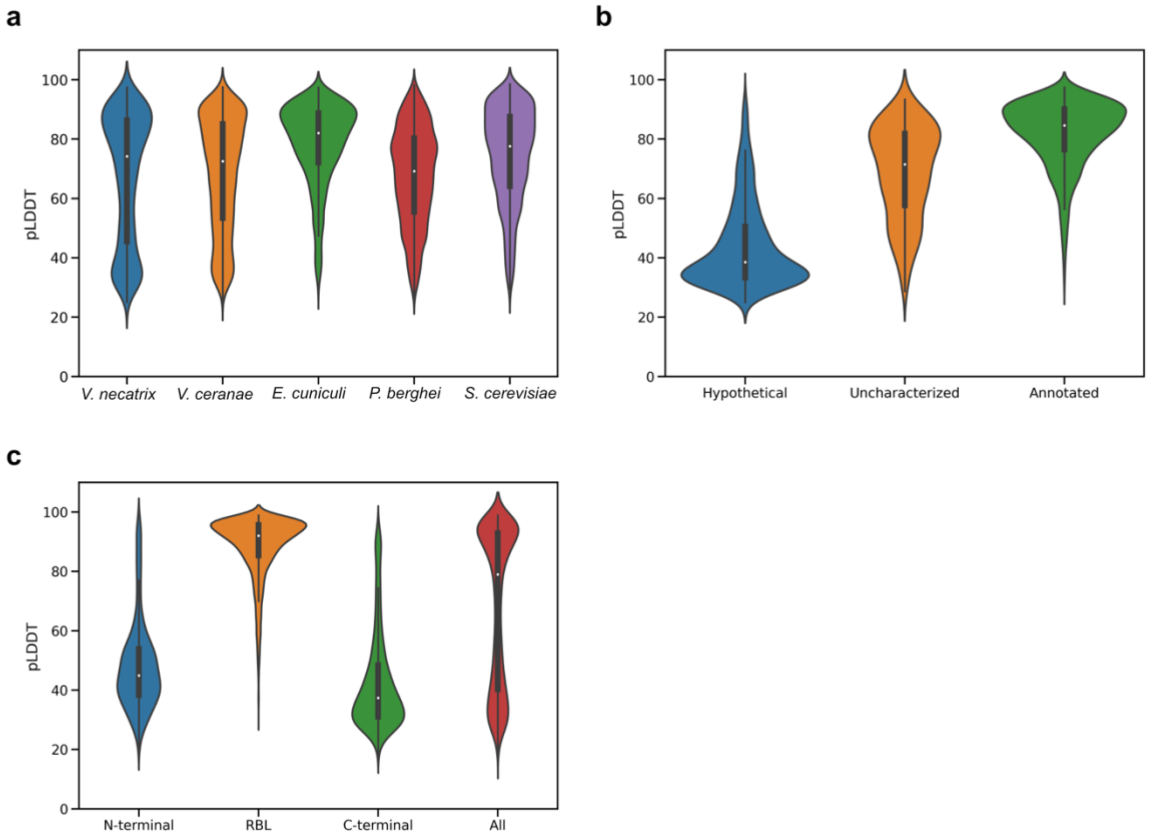

Supplementary Figure 4. AlphaFold2 pLDDT scores and structural prediction quality overall, of the final annotation and for the RBL protein regions. (**a**) Overall *V. necatrix* AlphaFold2 predictions, compared to other species (**b**) final annotations, (**c**) RBL proteins divided into N-terminus, RBL domain and C-terminus and whole RBL protein.

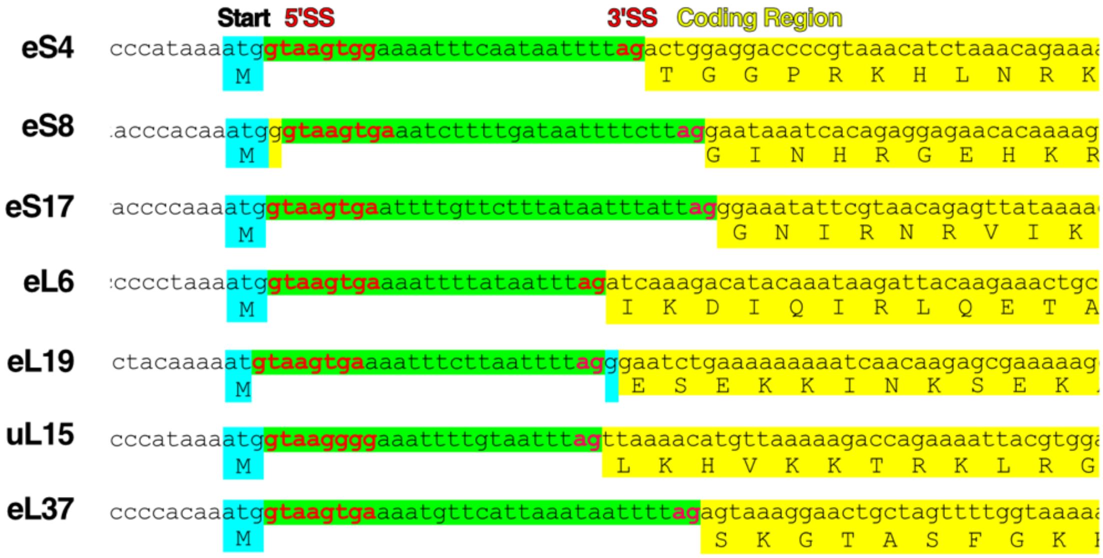

Supplementary Figure 5. Intron containing ribosomal proteins. The 5’ end of ribosomal protein genes with identified introns (green) are shown with both 5’ and 3’ splice sites (SS) indicated in red, if unambiguously identified. Potential start codon (blue) and coding region (yellow) are highlighted.

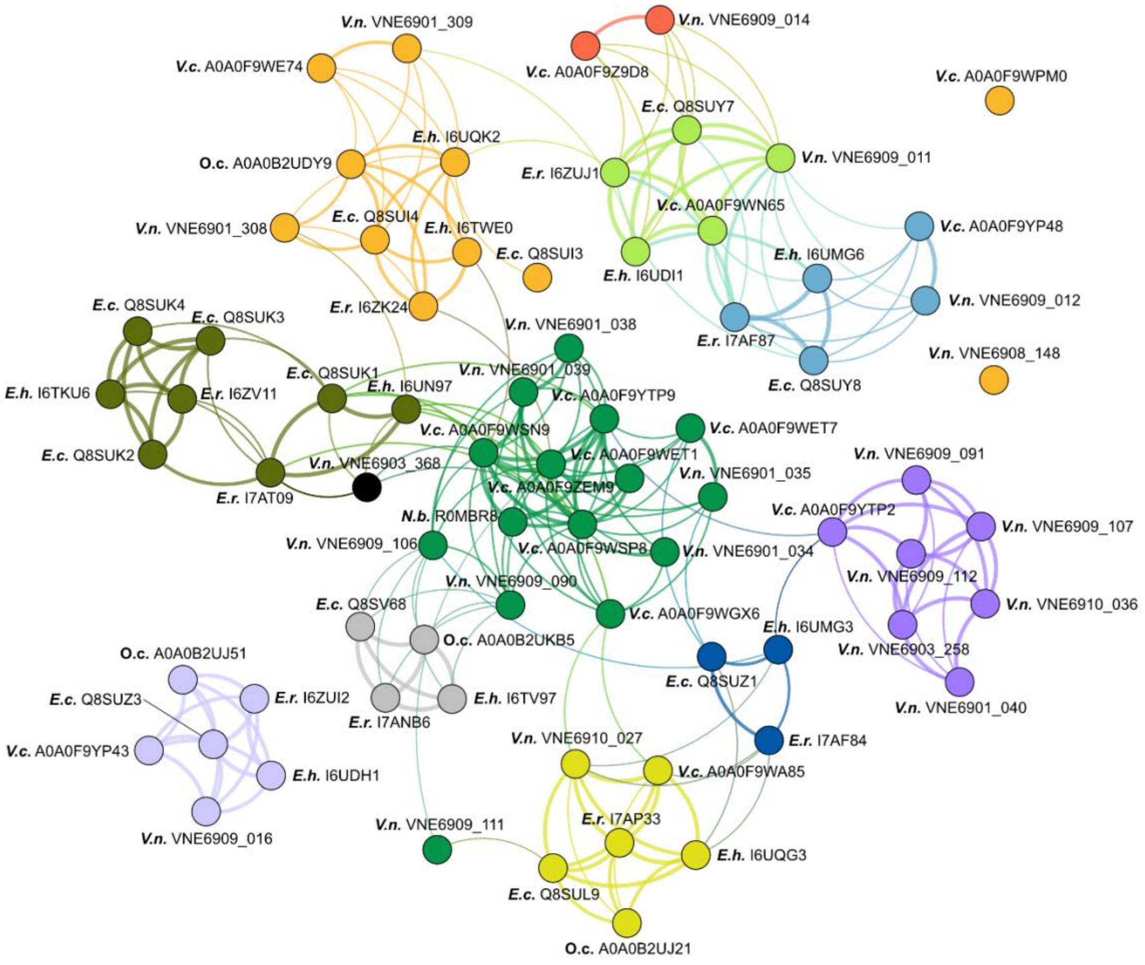

Supplementary Figure 6. Structural network of ricin B lectins in Nosematida shown with organism and gene IDs. RBL domain folds of all Nosematida RBL proteins identified in this study were predicted with AlphaFold and clustered according to structural similarity based on their TM score using Gephi. RBL domains are color-coded according to their RBL protein clade in **Figure 5a**. Each node represents one RBL domain, and connecting lines indicate the degree of structural relatedness.

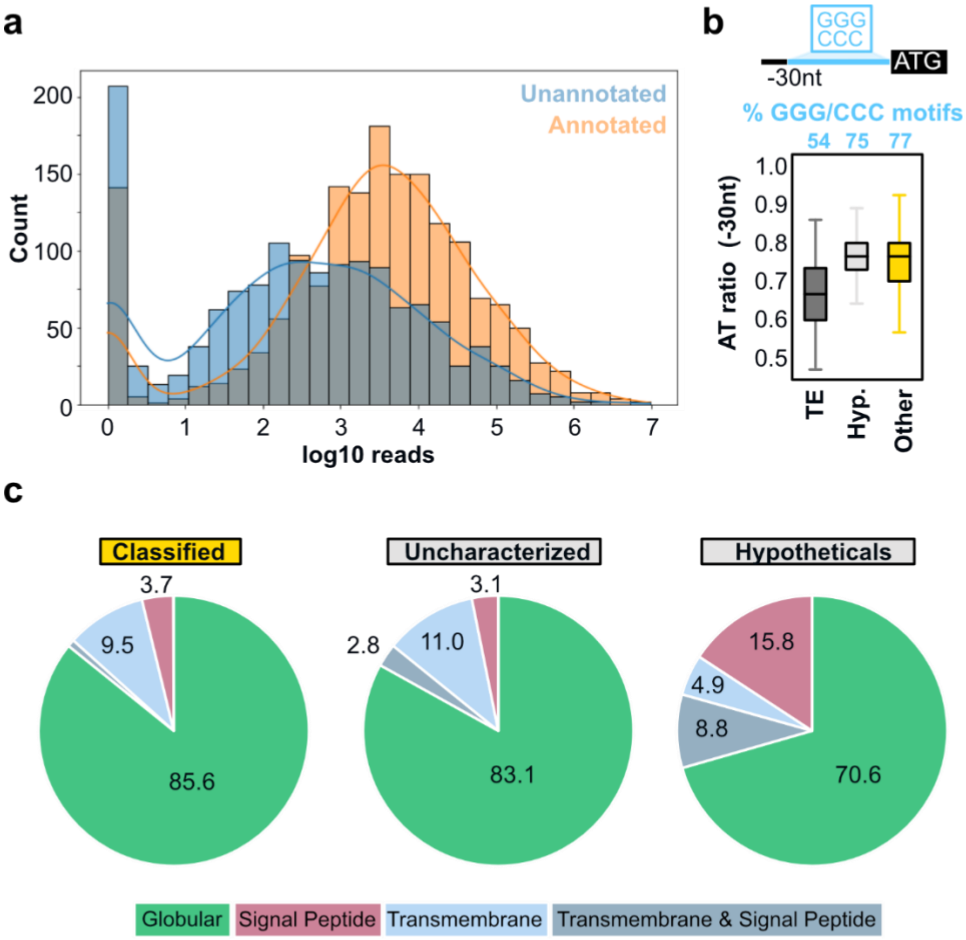

Supplementary Figure 7. RNA sequencing reads of annotated and unannotated genes, and protein features of hypothetical, uncharacterized, and classified proteins. (a) Distribution plot for RNA sequencing reads of annotated vs. unannotated genes. The bar plot is non-stacked, and the blue or grey bars correspond to unannotated genes and the orange bars correspond to annotated genes. (**b**) Percentage of CCC or GGG motifs (blue numbers) and histogram plot of the AT-ratio within the 30 nt upstream of the start codon for transposable elements (TE), hypotheticals, and all other proteins. (**c**) Pie plots of the classified, uncharacterized, and hypothetical gene groups with the percentage of globular (green), signal-peptide-containing (coral), transmembrane-containing proteins (light blue), and those with both TMD and SP (grey blue).
